# Expression Profile of Genes Related to the Th17 Pathway in Macrophages Infected by *Leishmania major* and *Leishmania amazonensis*: The Use of Gene Regulatory Networks in Modeling This Pathway

**DOI:** 10.3389/fcimb.2022.826523

**Published:** 2022-06-14

**Authors:** Leilane Oliveira Gonçalves, Andrés F. Vallejo Pulido, Fernando Augusto Siqueira Mathias, Alexandre Estevão Silvério Enes, Maria Gabriela Reis Carvalho, Daniela de Melo Resende, Marta E. Polak, Jeronimo C. Ruiz

**Affiliations:** ^1^ Programa de Pós-graduação em Biologia Computacional e Sistemas, Instituto Oswaldo Cruz, Fiocruz, Rio de Janeiro, Brazil; ^2^ Grupo Informática de Biossistemas, Instituto René Rachou, Fiocruz Minas, Belo Horizonte, Brazil; ^3^ Systems Immunology Group, Clinical and Experimental Sciences, Faculty of Medicine, University of Southampton, Southampton, United Kingdom; ^4^ Grupo Genômica Funcional de Parasitos, Instituto René Rachou, Fiocruz Minas, Belo Horizonte, Brazil

**Keywords:** *Leishmania*–macrophage interaction, gene regulatory network (GRN), expression profiling, Th17, immune modulation

## Abstract

*Leishmania amazonensis* and *Leishmania major* are the causative agents of cutaneous and mucocutaneous diseases. The infections‘ outcome depends on host–parasite interactions and Th1/Th2 response, and in cutaneous form, regulation of Th17 cytokines has been reported to maintain inflammation in lesions. Despite that, the Th17 regulatory scenario remains unclear. With the aim to gain a better understanding of the transcription factors (TFs) and genes involved in Th17 induction, in this study, the role of inducing factors of the Th17 pathway in *Leishmania*–macrophage infection was addressed through computational modeling of gene regulatory networks (GRNs). The Th17 GRN modeling integrated experimentally validated data available in the literature and gene expression data from a time-series RNA-seq experiment (4, 24, 48, and 72 h post-infection). The generated model comprises a total of 10 TFs, 22 coding genes, and 16 cytokines related to the Th17 immune modulation. Addressing the Th17 induction in infected and uninfected macrophages, an increase of 2- to 3-fold in 4–24 h was observed in the former. However, there was a decrease in basal levels at 48–72 h for both groups. In order to evaluate the possible outcomes triggered by GRN component modulation in the Th17 pathway. The generated GRN models promoted an integrative and dynamic view of *Leishmania*–macrophage interaction over time that extends beyond the analysis of single-gene expression.

## Introduction

Leishmaniasis is a complex of infectious diseases caused by different species of the protozoan parasite *Leishmania* (Kinetoplastida and Trypanosomatidae) that represent one of the major public health problems in developing countries, with 12 million cases and an incidence of 0.7–1.0 million new cases annually, according to the WHO (WHO, 2020). Depending on the *Leishmania* species involved, the disease can comprise two main clinical forms: visceral leishmaniasis or cutaneous leishmaniasis (Burza et al., 2018). Between both clinical forms, the former has the highest prevalence and incidence and is caused by different *Leishmania* species, including, but not restricted to, *Leishmania major*, *Leishmania amazonensis*, *Leishmania braziliensis*, *Leishmania mexicana*, and *Leishmania peruviana* (Martinez and Petersen, 2014). In humans, macrophages are the final host cells for *Leishmania* parasites, playing a dual role in the progression of the infection, responsible for control or exacerbation of the immune response that culminates in different clinical manifestations (Tomiotto-Pellissier et al., 2018). Infections by *Leishmania* sp. promote different types of immune responses, creating a microenvironment of cytokines and chemokines, which is a determinant of the establishment or not of the disease. In this context, T helper cell responses have an important role, through different T cell subsets such as Th1, Th2, Th17, and Tregs (Gonçalves-de-Albuquerque et al., 2017). The Th1 response drives an increase of CD4+ T cells with the production of interferon-γ, interleukin (IL) 2 and IL-12 that stimulate macrophages to fight against infected cells (Isnard et al., 2012; Mutiso et al., 2013). However, if the immune response is Th2 type, IL-4 and IL-10 are produced, which act in inhibiting the activation of macrophages, consequently promoting the aggravation of the disease (Isnard et al., 2012; Liu and Uzonna, 2012; Mutiso et al., 2013; de Paiva et al., 2015). Lastly, Th17 cells may play an essential role in protecting against certain extracellular pathogens and have been demonstrated to influence the balance between inflammatory and anti-inflammatory cytokines, which must be orchestrated in the course of infection to guide a successful effector response to the intracellular *Leishmania* protozoa (Gonçalves-de-Albuquerque et al., 2017). Particular interest in the Th17 type remains focused on its main cytokine, IL-17. Although it is also produced by other cells including CD8+ T cells and neutrophils, IL-17 is mostly produced by Th17 cells. Thus, the effects of Th17/IL-17 are still unclear, as disease-promoting and protective responses have both been attributed to their influence. In this scenario, the Th17 cell activities in the context of the different clinical forms of leishmaniasis, interaction with regulatory cytokines, and host particularities remain to be explored (Anderson et al., 2009, 17; Liu and Uzonna, 2012, 17; Gaffen et al., 2014; Bartlett and Million, 2015; Gonçalves-de-Albuquerque et al., 2017, 17; Tomiotto-Pellissier et al., 2018, 17).

The response of infection, in macrophages, occurs due to the activation of different transcription factors (TFs), such as STAT1, IRF, NF-κB, and STAT6 (Bichiou et al., 2021; Murray, 2017).

Although many reviews have discussed the cellular immune responses against the different forms of leishmaniasis (Pitta et al., 2009; Hu et al., 2010; Gonzalez-Lombana et al., 2013; Gonçalves-de-Albuquerque et al., 2017; Banerjee et al., 2018) and the roles of Th17/IL-17 in infectious and non-infectious diseases, their role in the immunopathogenesis of leishmaniasis remains unclear (Gonçalves-de-Albuquerque et al., 2017).

Some studies (Vargas-Inchaustegui et al., 2008; Boaventura et al., 2010, Castilho et al., 2010; Katara et al., 2012; Gonzalez-Lombana et al., 2013; Katara et al., 2013; Bartlett and Million, 2015; Banerjee et al., 2018) have suggested that high levels of IL-17 may play an important pro-inflammatory role in leishmaniasis development and as such should be considered an important target for the disease immunotherapy.

An alternative to better understanding the role of these elements of the immune response in the immunopathogenesis of leishmaniasis would be through the use of gene regulatory networks (GRNs). GRNs can be defined as mathematical and computational models capable of describing the logic underlying the regulatory occurrences among interacting genes while a specific cell program is operating. The fundamental challenge that GRNs address is the translation between the diverse information patterns presented by different types of biological macromolecules into biologically significant knowledge. This knowledge is directly associated with the time-dynamic processes necessarily underlying any kind of observable biological behavior (Bansal et al., 2006; Anitha et al., 2016; de Luis Balaguer et al., 2017; Banf and Rhee, 2017; de Luis Balaguer et al., 2017; Peter and Davidson, 2017; Delgado and Gómez-Vela, 2019). A key element in the modeling process of GRNs is the identification of molecular regulators associated with the activation and control of gene expression, to which TFs are considered essential. They modulate the rate of expression of genes by inhibiting or enhancing the gene’s transcription rates and, in addition, interact with regulatory elements to generate specific gene expression patterns. Additionally, TFs are themselves the products of gene expression, serving as a source of feedback for the regulation of genetic networks (Steele et al., 2009; Chandrasekaran et al., 2011; Ghedira et al., 2011; Sinha et al., 2020).

Different approaches used for modeling GRNs are described in the literature (Miyagi et al., 2002; Hardy and Robillard, 2004; Anitha et al., 2016; Livigni et al., 2016; Pennisi et al., 2016; Singh et al., 2020). These approaches include differential equations, stochastic simulations, flux balance analysis, graphical models, and Petri nets (PNs). PNs are an *in silico* mathematical approach that provides a visual-aided network-oriented modeling process based on nodes and edges to represent places and transitions, with visual feedback affording interpretation by a broad audience and can be applied to describing distributed systems, including biological ones (Hardy and Robillard, 2004; Livigni et al., 2016). In the context of PNs, places represent molecules (proteins, mRNA, and complexes), transitions represent possible processes (binding, phosphorylation, and inhibition, including reversibility, among others), and tokens represent concentrations.

On this basis, we decided to use GRNs to evaluate the dynamic of the infection profile correlated with the modulation of Th17 immune response in cutaneous leishmaniasis through the utilization of data from transcriptome sequencing of macrophages infected with *L. major* and *L. amazonensis* associated with data experimentally validated in the literature.

## Materials and Methods

### Dataset Selection

A total of 66 samples from the study SRP062278 were performed by Fernandes et al., 2016 (Fernandes et al., 2016). The data were downloaded from the Sequence Read Archive (SRA) database using SRA prefetch and converted to fastq files using fastq-dump, available in SRA toolkit version 2.10.2. All mRNA paired-end libraries with 100 bp were sequenced using Illumina Hiseq 1500 platform. The study SRP062278 comprises a time-series experiment with four different time points (4, 24, 48, and 72 h post-infection (hpi) of human macrophages infected with *L. amazonensis* (IFLA/BR/67/PH8), human macrophages infected with *L. major* (clone VI, MHOM/IL/80/Friedlin), uninfected human macrophages, and macrophages containing latex beads.

### Data Preprocessing

The sequence quality of the downloaded data was assessed using the FastQC program (https://www.bioinformatics.babraham.ac.uk/projects/fastqc/) and visualized using MultiQC reporting tool (Ewels et al., 2016).

As references, the human transcriptome data in FASTA and GTF format was obtained from the Ensembl database (ftp://ftp.ensembl.org/pub/release-99/), release 99. FASTA and GFF files with transcript sequences and annotations from *L. amazonensis* MHOM/BR/71973/M2269 and *L. major* Friedlin strain were downloaded from TriTrypDB (https://tritrypdb.org/tritrypdb/), release 46 (de Azevedo et al., 2010). The transcripts sequences of *L*. *amazonensis* MHOM/BR/71973/M2269 were used as a reference due to the absence of sequencing information of *L. amazonensis* IFLA/BR/67/PH8 in public databases.

### Transcriptome Quantification and Differential Gene Expression Analyses

Kallisto (version 0.46.1) (Bray et al., 2016) was used to generate the transcriptome indexes for human and *Leishmania* sp. data. All samples were pseudoaligned against the references and quantified using Kallisto quant, using bias correction parameter and 100 as the bootstrap number. The differential gene expression analysis was performed in R using Sleuth (version 0.30.0) (Pimentel et al., 2017), Limma (version 3.42.1) (Law et al., 2018), and edgeR (version 3.28.0) (Robinson et al., 2010) packages. In order to identify differentially expressed (DE) genes, an adjusted *p*-value of less than 0.05 was set as a threshold to define the significance.

### Network Analysis

The network analysis was performed using Graphia Pro, available in BioLayout Express3D (Theocharidis et al., 2009), where a gene correlation matrix was calculated for all DE human genes fulfilling the criteria mentioned above and considering that each column of data represents one sample and each row of data represents one gene.

A network graph of the data was generated with a Pearson’s correlation coefficient of r ≥ 0.85 using TMM normalization. The network graph was then clustered into groups of genes sharing similar expression profiles using the MCL (Li, 2003) algorithm with an inflation value of 1.7. All the clusters were analyzed considering the differences in the expression profiles between the conditions and functionally annotated using ToppGene tool (Chen et al., 2009) and David (Dennis et al., 2003) software.

With the use of the information related to the clusters functional annotation, the most relevant pathways in *Leishmania* infection were evaluated, and the Th17 pathway was selected (**Supplementary Table 1**).

### Model Assembly

In order to identify components for the Th17 GRN, a systematic search in PubMed was performed. Of the returned papers, 327 were identified describing the IL-17 pathway and its TFs. The experimental findings within each listed reference paper were analyzed and categorized as the input information for the network into a) partner 1 (a gene that acts by somehow regulating a second gene); b) partner 2 (a gene that undergoes some type of induction/inhibition by partner 1); c) TFs that interact with partners 1 and 2; d) cell type in which this interaction was observed; e) type of interaction; and f) induced biological process and outcome of the interaction between partners 1 and 2 (activation of other genes, induction of cytokines or chemokines). To be included in the network diagram, all the identified interactions had to be confirmed by at least two different publications. This information was categorized as the network components: input node, transmission node, an output node, and mode of interaction (**Supplementary Table 2**).

In the model, edges, connecting nodes, and transitions determine the direction of information flow, representing the progress of the biological process over time (time-blocks). Each infection time in the experimental design represents a time-block that was used for the simulation. Thus, the time-blocks were defined as a) 4 hpi (time-block 0–25), b) 24 hpi (time-block 26–50), c) 48 hpi (time-block 51–75), and d) 72 hpi (time-block 76–100).

### IL-17 Gene Regulatory Network Model Parametrization and *In Silico* Simulations

The network diagram was constructed using yEd Graph Editor following the mEPN notation, as described by Livigni et al. (2018), and saved as GraphML files (Livigni et al., 2016; O’Hara et al., 2016).

Prior to performing a simulation, a model was parameterized through the places representing the TFs at the beginning of the network. For this parametrization, the normalized read count values obtained from the RNA-seq data were used to parametrize the input tokens that correspond to TFs identified in the literature review that interact with one or more genes. This resulted in the modeling of three networks: the first considering the uninfected macrophages, the second considering macrophages infected with *L. amazonensis*, and the third as macrophages infected with *L. major*.

GraphML files were loaded into the BioLayout Express 3D, and a parser translated the diagrams drawn using the mEPN scheme. Once the files were imported, the conditions for stochastic PN (SPN) simulation were set (i.e., mode of stochasticity, number of runs, and defined time-blocks).

Each time point in the experimental design represents an interval of the time-blocks used for the simulation. All the measured read count averages for the TFs identified, at 4 hpi (0–25 time-block), 24 hpi (26–50 time-block), 48 hpi (51–75 time-block), and 72 hpi (76–100 time-block), were converted into parameterization values for each entry node in the GRN. Simulations were conducted in Graphia Pro using 100 time-blocks, 500 runs, uniform distribution in SPNs, and distribution and consumptive transition in SPN transition type.

During the simulation, some inhibitions and blocking edges were also made in the constructed models in order to observe the impact of this alteration on the outcome (Th17 response).

### Statistical Analyses

In order to compare the TFs in each time point for each type of infected macrophage, the statistical analysis was performed using mixed ANOVA, with a general linear model (α = 0.05). Sphericity was evaluated using Mauchly’s test (α = 0.05) and/or cases where it was not verified; the Greenhouse–Geisser correction was performed to verify the effect of the independent variables and their interactions on the expression value of each of the factors individually. To locate the observed differences, a *post-hoc* analysis with Bonferroni correction was conducted, also with a significance level of 0.05.

## Results

### Transcriptome Quantification and Differential Gene Expression Analyses

Approximately 90% of reads were mapped against the reference transcriptomes. The samples SRR2163291 (macrophage containing beads at 48 hpi) and SRR2155160 (macrophage infected with *L. major* at 24 hpi) were identified as outliers and removed from the analyses due to the observation of distinct profiles in the principal component analysis.

Considering the applied cutoff to define significance, for the macrophage infected with *L. major* dataset when compared to uninfected macrophages, for each time-point (24, 224, 48, and 72 hpi), a total of 3,082, 1,389, 1,193, and 642 DE genes were identified, respectively.

For the dataset comprising the macrophages infected with *L. amazonensis*, when compared to uninfected macrophages, a total of 2,452, 1,332, 301, and 327 DE genes were found for each time-point (24, 224, 48, and 72 hpi, respectively). The results are depicted in **Table 1**.

**Table 1:** Differentially expressed genes.

Time point	Macrophages infected with Leishmania major*		Macrophages infected with Leishmania amazonensis*	
	Upregulated	Downregulated	Upregulated	Downregulated
4 h post-infection	1,604	1,478	1,407	1,045
24 h post-infection	700	698	652	680
48 h post-infection	689	504	84	217
72 h post-infection	361	281	192	135

*When compared to uninfected macrophages.

The most expressed genes in infected macrophages were functionally annotated. For the biological process category, the main ontologies identified in both datasets (macrophages infected with *L. major* and macrophages infected with *L. amazonensis*) were a cellular response to the stimulus caused by DNA damage (GO:0006974), DNA repair (GO:0006281), DNA metabolic process (GO:0006259), repair (GO:0006260), and modifications in lysines (GO:0018205). Considering the category of molecular function, the main ontologies identified were related to insertion or deletion of DNA bases (GO:0032135), DNA binding (GO:0003684), and DNA-dependent ATPase activity (GO:0008094). For the cellular component category, ontologies related to chromatin (GO:0000785) and centrosome (GO:0005813) were identified.

The most overrepresented in the dataset was grouped based on semantics, and it was observed that most of the functions found were related to changes in the cell cycle, stress response, DNA repair mechanisms, protein modification processes, and diversification of immunological receptors.

### Differentially Expressed Gene Clustering and Annotation

A network graph of the data was generated with a Pearson’s correlation coefficient of r ≥ 0.85 using TMM normalization in the read count table and clusterized using the MCL algorithm.

As result, a total of 57 clusters were obtained, but in only 30 clusters was it possible to identify significant alterations in the expression of a set of genes in macrophages infected with *L. majo*r and *L. amazonensis* when compared to uninfected macrophages (**Supplementary Table 1**).

The genes comprising the selected clusters were functionally annotated, which allowed the identification of many immunological pathways such as tumor necrosis factor (TNF) signaling pathway, *NF-kappa B* signaling pathway, cytokine–cytokine receptor interaction, Chagas disease (*American trypanosomiasis*), MAPK signaling pathway, toll-like receptor signaling pathway, IL-10 signaling pathway, and TH17/IL-17 signaling pathway.

Considering the pathways found in the annotation, a literature review was conducted in order to identify the most relevant pathways for *Leishmania* infection. In the literature review, using the search terms depicted in **Supplementary Table 2**, a total of 22 interactions were found considering the TFs, and a total of 73 interactions considering the genes in the IL-17 pathway were identified in at least 2 papers to be considered for the network model. All this information was categorized as network components, receiving the nomenclature of a) input node (for TFs) (n = 12); b) transmission node: genes that are activated during the course of infection (n = 22); c) output node: genes that are activated for a given immune response (n = 16); and d) mode of interaction: how these genes interact, whether through activation, binding, inhibition of phosphorylation.

### Model Assembly and Parametrization

**Figure 1:**
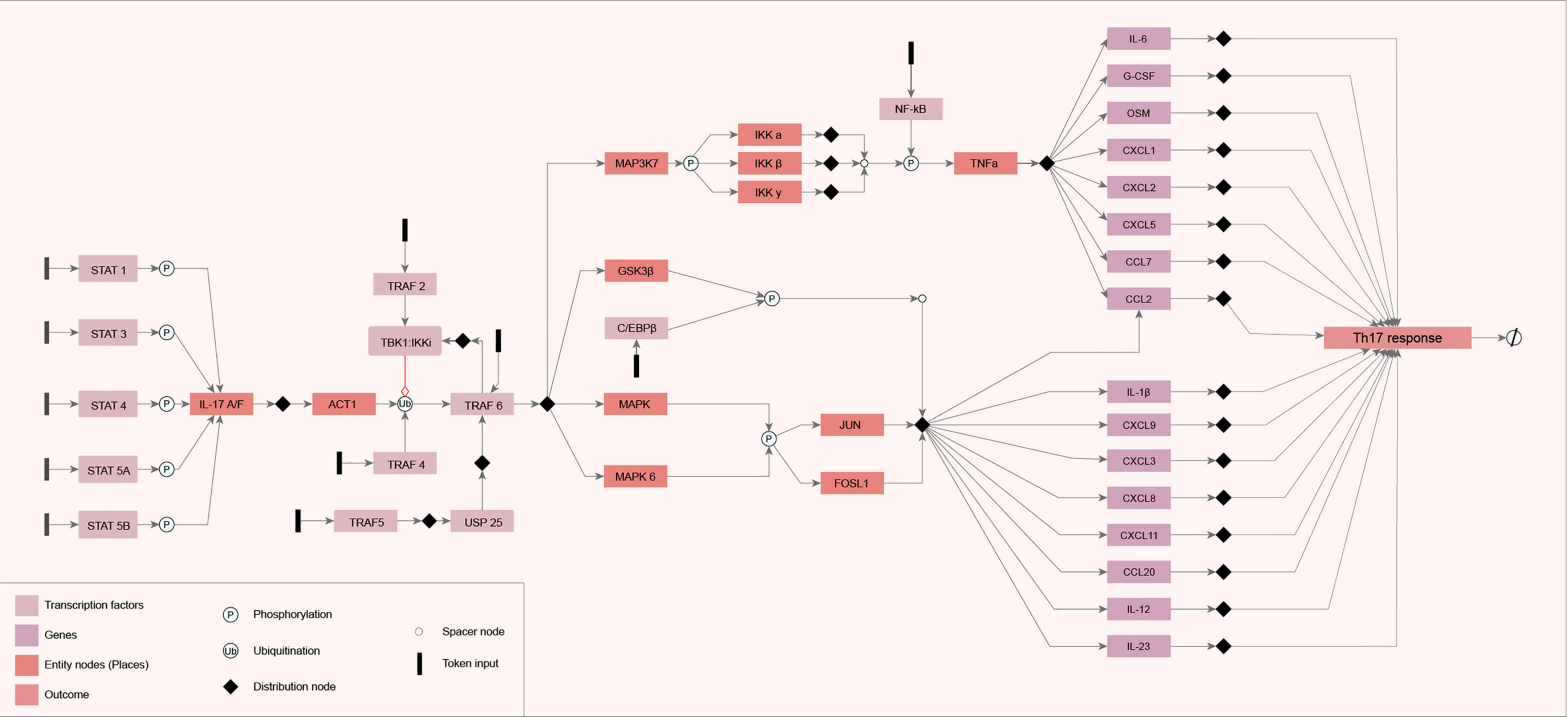
Diagram of the constructed network. The network diagram was constructed using the yEd Graph Editor according to the mEPN notation, described by Livigni et al. (2018), using the information obtained from the search carried out in the literature. The components (genes) in the IL-17 gene regulatory network (GRN) are represented by rectangles connected by arrows that indicate the molecular interactions found during the literature review step. System start nodes (transcription factors) are represented by colored rectangles in black. The GRN output signal is represented in the rectangle on the right side of the diagram.

All the information categorized in the literature review was used to model the IL-17 network diagram. The components (genes) in the IL-17 GRN are represented by rectangles connected by arrows, which indicate the molecular interactions found during the literature review step. System start nodes (TFs) are represented by colored rectangles in black. The GRN output signal (Th17 response) is represented in the rectangle on the right side of the diagram (**Figure 1**). We observed that the flow of information starts from the activation of TFs and follows all the models of the genes and the interactions between them. All these interactions converge for the activation of cytokines, chemokines, and other ILs, such as IL-6 and IL-12.

As a result of model parameterization, we identified the TFs involved in the IL-17 pathway and calculated the average of expression counts for each time-block evaluated (**Table 2**).

**Table 2:** Read count average for each transcription factor in IL-17 pathway for each time-block defined interval.

Transcription factor	Time-block	Read count average		
		Uninfected macrophage	Macrophage infected with *Leishmania major*	Macrophage infected with *Leishmania amazonensis*
STAT1	0–25	5,877.61	5,372.16	4,952.21
	26–50	6,754.95	5,297.05	9,402.04
	51–75	4,595.94	7,787.30	7,038.73
	76–100	4,764.34	6,128.42	2,346.24
STAT4	0–25	103.36	316.41	219.78
	26–50	16.21	22.79	56.48
	51–75	9.46	9.43	28.13
	76–100	10.44	7.51	9.38
STAT5A	0–25	1,377.02	1,127.25	887.90
	26–50	1,454.16	990.51	1,214.90
	51–75	1,545.59	1,210.43	1,210.77
	76–100	1,581.53	1,422.85	403.59
STAT5B	0–25	524.35	407.32	429.26
	26–50	532.86	319.05	405.40
	51–75	592.85	464.02	472.34
	76–100	618.74	502.93	157.45
TRAF4	0–25	43.62	54.67	55.00
	26–50	25.99	28.18	48.88
	51–75	30.51	63.88	55.27
	76–100	27.78	48.00	18.42
TRAF6	0–25	442.61	654.83	652.17
	26–50	359.55	268.30	374.58
	51–75	382.51	338.58	368.61
	76–100	404.66	414.34	122.87
NFKB1	0–25	1,184.41	5,017.29	4,894.69
	26–50	681.48	694.29	1,270.40
	51–75	436.75	580.69	695.09
	76–100	439.57	460.40	231.70
TRAF3	0–25	1,635.59	2,203.29	2,435.34
	26–50	1,600.42	1,746.89	1,915.48
	51–75	1,291.95	1,465.59	1,686.70
	76–100	1,372.07	1,247.54	1,487.19
TRAF5	0–25	561.71	1,832.44	1,460.42
	26–50	508.41	401.60	436.62
	51–75	618.41	594.85	606.95
	76–100	642.19	634.43	631.53
NFKBIZ	0–25	280.17	661.13	880.30
	26–50	421.33	408.78	677.47
	51–75	208.93	241.42	381.54
	76–100	179.65	173.16	231.80
CEBPD	0–25	422.55	184.11	163.55
	26–50	157.70	51.71	47.14
	51–75	166.76	73.86	51.91
	76–100	145.51	94.86	41.46
CEBPB	0–25	4,759.21	10,652.21	11,066.34
	26–50	2,663.82	5,337.31	5,726.38
	51–75	1,904.76	4,190.46	4,000.16
	76–100	1,737.65	3,264.60	3,344.22
AP-1	0–25	2,401.65	8,360.52	9,936.31
	26–50	1,359.71	2,622.15	3,633.69
	51–75	1,260.27	1,768.60	2,268.36
	76–100	1,272.11	2,044.06	2,052.19

**Figure 2:**
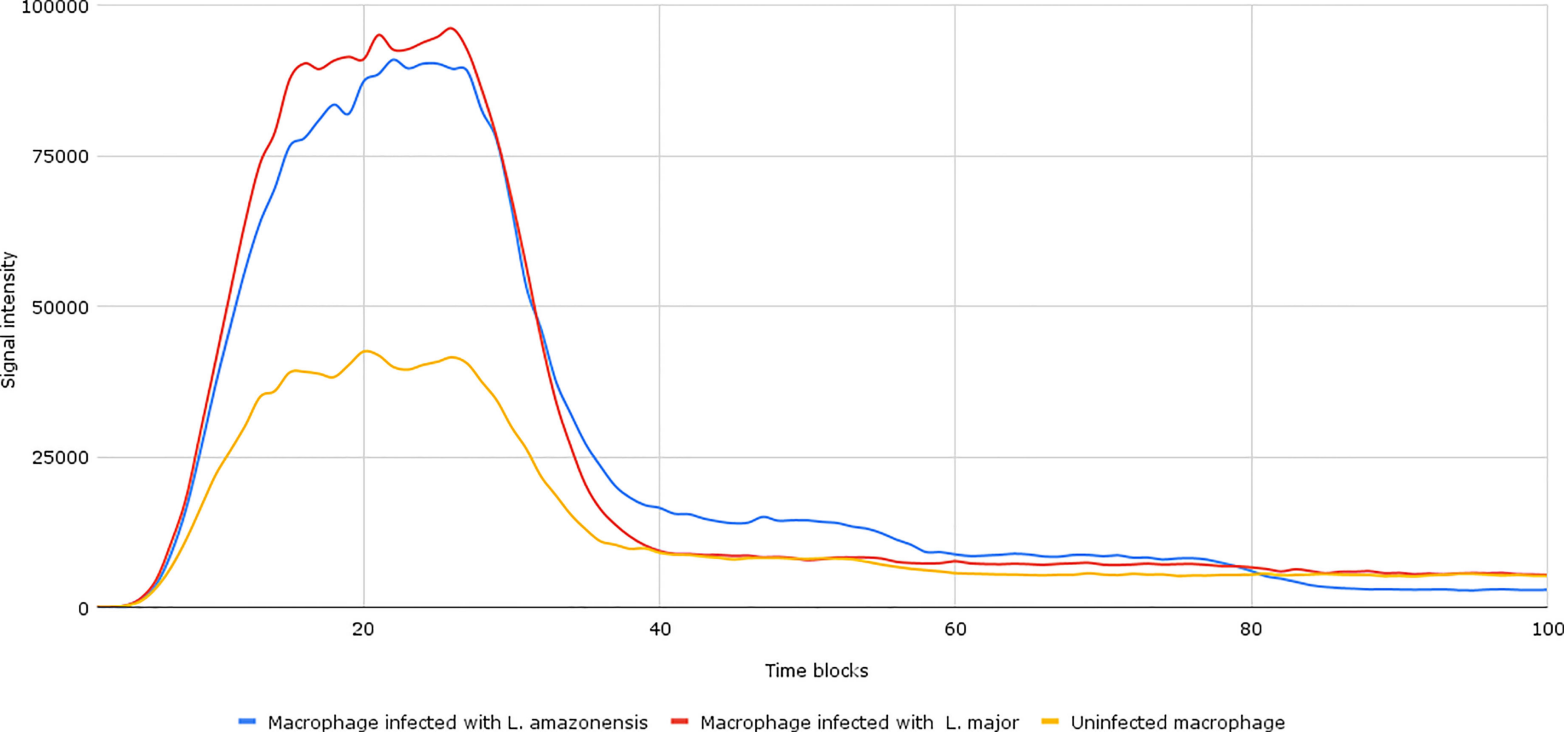
Accumulation token result of the initial network simulation. Under normal conditions, as can be seen in the yellow lines (uninfected macrophages), the final response signal intensity is about 2–3 times lower than when compared to samples of *Leishmania major*- and *Leishmania amazonensis*-infected macrophages (represented by the red and blue lines, respectively). To be generated, the initial information flow starts with the activation of transcription factors (input nodes). These factors interact with a set of genes (transmission node) through processes (mode of interaction). These processes are responsible for intensifying or reducing the intensity of the signal generated in response.

After the establishment of parameterization, we performed the network simulations. Under normal conditions, as can be seen in **Figure 2**, the signal intensity is about 2–3 times lower than when compared to samples of macrophages infected by *L. major* and *L. amazonensis*.

**Figure 3:**
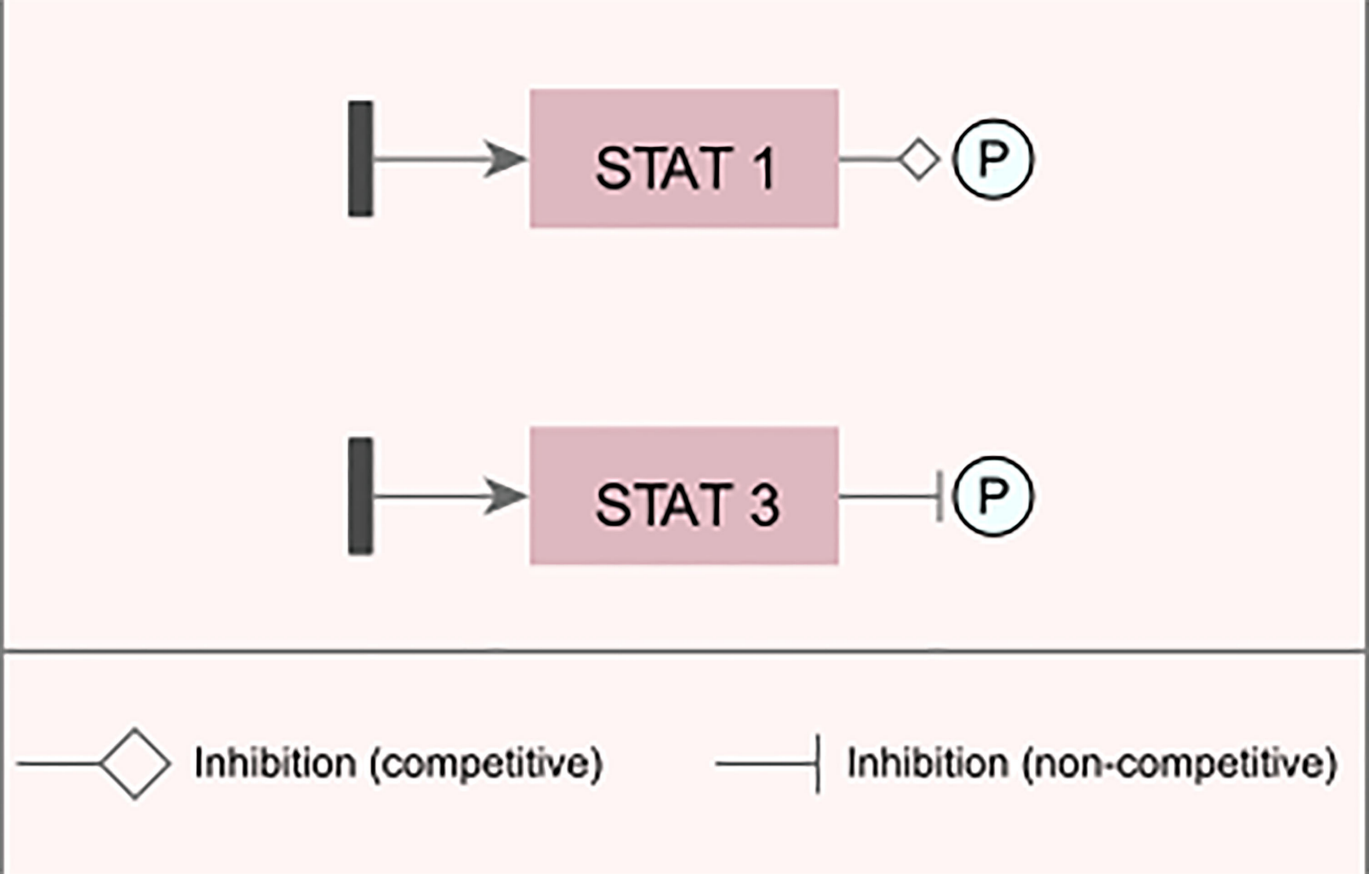
Examples of changes inserted into the network. Examples of representation of inhibitory and/or blocking changes that were inserted into the modeled network.

After the network parameterization and simulation, inhibition points on the edges between certain interactions were inserted in order to observe how the signal intensity changed based on the modifications in the model (**Figure 3**).

For the transcription factor STAT1 (signal transduction and activator of transcription 1), an edge change was inserted, which reduces the intensity in which phosphorylation is performed on the target gene for its activation, while in the STAT3 factor, a blocking edge was inserted, which completely inhibits the signal generated by this TF; i.e., it does not phosphorylate the target gene.

**Figure 4:**
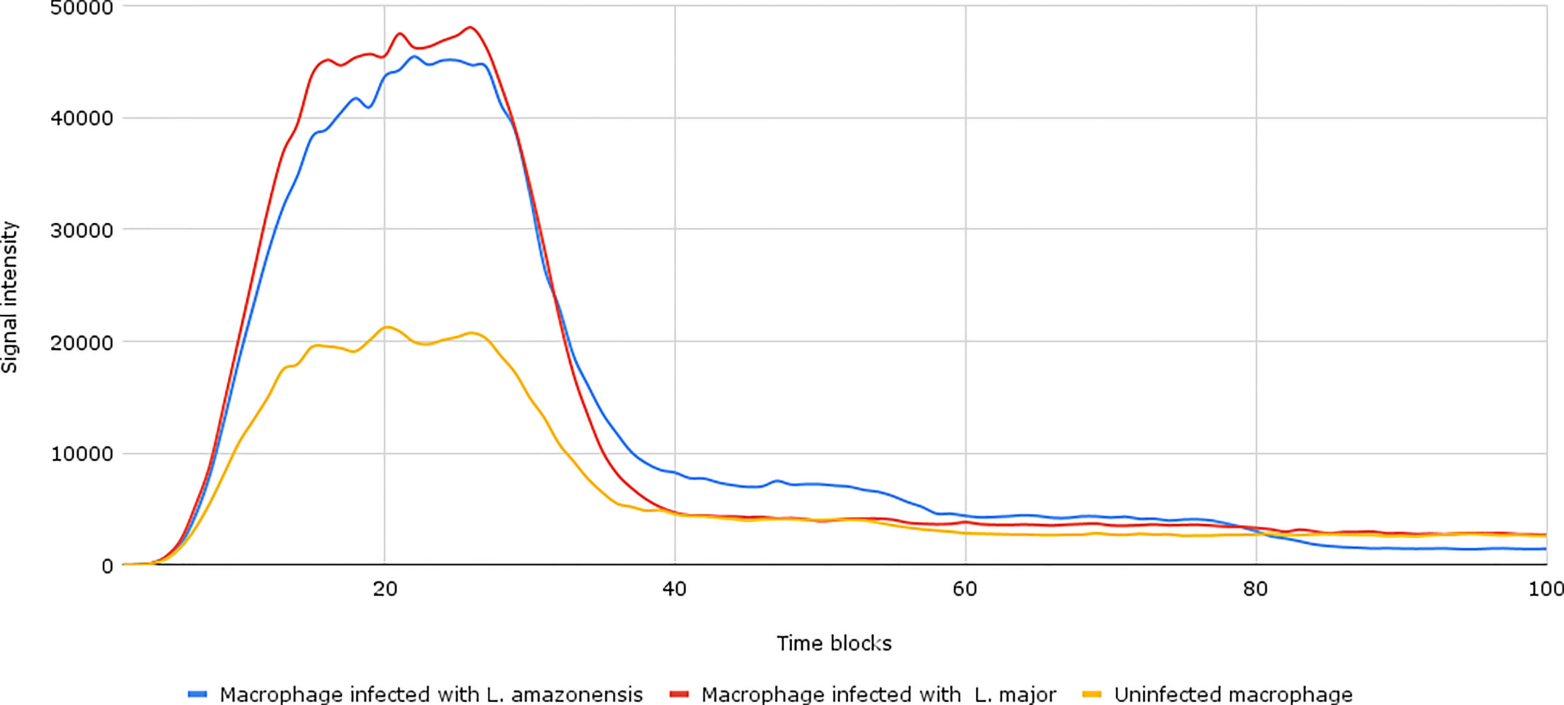
Result of the network simulation with changes in transcription factors. Under normal conditions (uninfected macrophages), as can be seen in yellow lines, the intensity of the final response signal is lower than when compared to infected macrophages with *Leishmania major* and *Leishmania amazonensis* represented in red and blue lines, respectively.

In addition to these two modifications in the TFs STAT1 and STAT3, partial inhibitory modifications were also inserted in CEBPβ (CEBPB-CCAAT/enhancer-binding protein beta) and NF-kβ (nuclear factor kappa-light-chain-enhancer of activated B cells). These modifications caused differences in the intensity of the signal generated (**Figure 4**), although they were not sufficient for a complete blockade of the Th17 response induction in infected macrophages when compared to uninfected macrophages.

After the inhibition of selected TFs, we noticed a decrease in the expression of CxCL2 and CCL7 in macrophages infected with *L. major.* For *L. amazonensis*, a decrease in CxCL8 and CXCL9 expression levels was also observed. The addition of inhibitory points in NF-kβ also causes a decrease in the potential to induce IL-12 and IL-23 levels. However, even with the inhibition of TFs, the gene expression related to the IL-17 pathway on infected macrophages did not equalize with non-infected macrophages.

**Figure 5:**
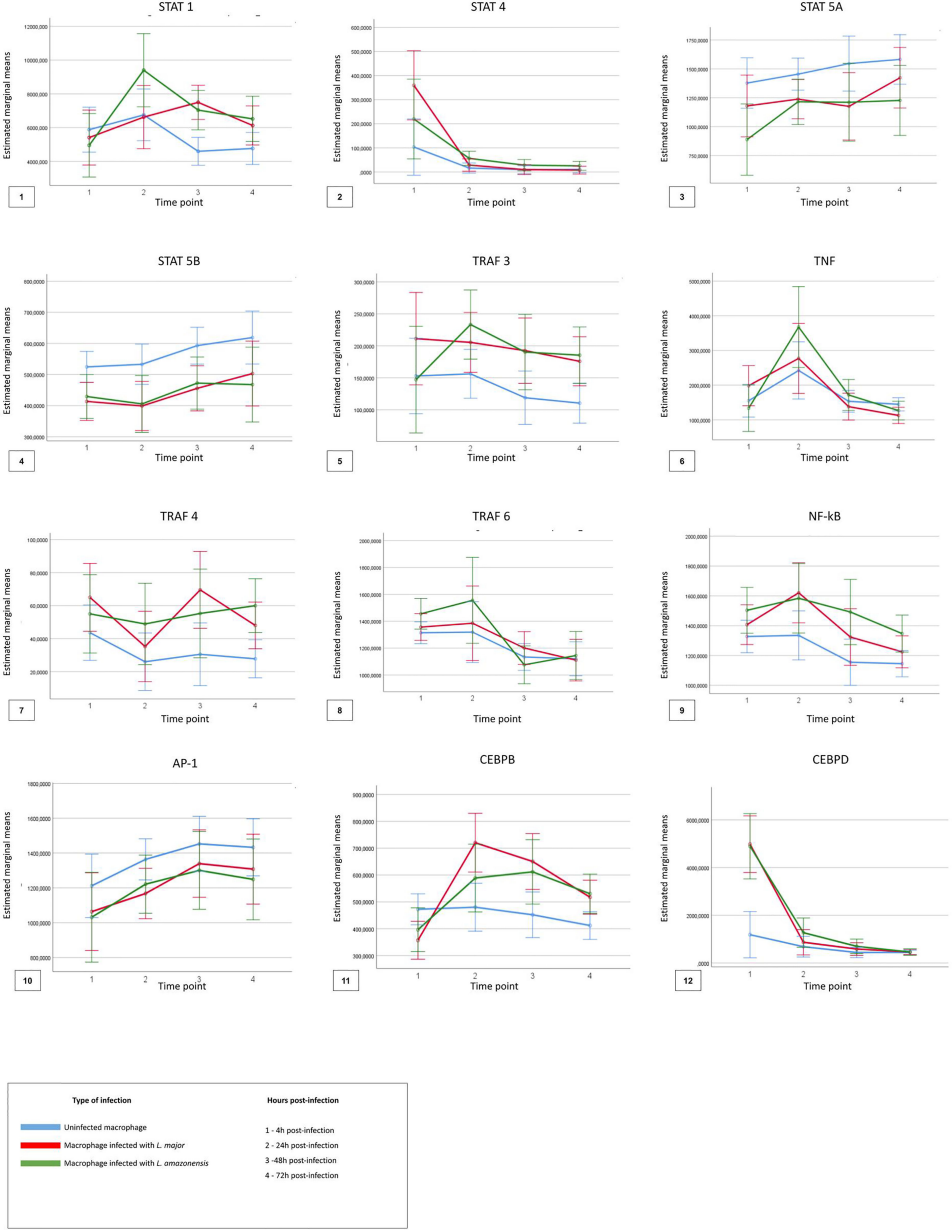
Descriptive analysis of expression dynamics for the transcription factors in the four time points post-infection. The time points 1, 2, 3, and 4 represent each hour post-infection in the kinetics, respectively, 4 h post-infection, 24 h post-infection, 48 h post-infection, and 72 h post-infection. The blue lines represent uninfected macrophages, the red lines represent macrophages infected with *Leishmania major*, and the green lines represent macrophages infected with *Leishmania amazonensis*.

For the analysis considering the TF comparisons between the time points (4, 24, 48, and 72 hpi) and across the macrophage’s type (uninfected, infected with *L. major*, and infected with *L. amazonensis*), **Figure 5** shows the descriptive analysis of the dynamics of expression of the factors in the four time points.

Significant differences (p < 0.05) in the expression of only STAT1, STAT4, TRAF6, and NFKB1 factors were observed for the different types of macrophages, at different times of infection. The expression of STAT 1 and STAT 4 was more associated in the early points of the infection (4–24 hpi) in macrophages infected with *L. amazonensis* and macrophages infected with *L. major*, respectively. An increase of expression for NF-κB was also observed in the time of 4 hpi for all infected macrophages when compared to uninfected macrophages.

## Discussion

*Leishmania* parasites are capable of modulating the immunological response and many signaling pathways on host cells in order to promote survival and infection (Mandlik et al., 2016). The key points for a parasite infection are related to the ability to escape from the macrophage leishmanicidal mechanisms and modulate the immune system in order to survive (Ovalle-Bracho et al., 2015; Akhoundi et al., 2017). It is known that all these cellular mechanisms comprise a set of signaling events that result in the modulation of TFs, which lead to different gene expression patterns (Contreras et al., 2010; Grünebast and Clos, 2020; Lecoeur et al., 2020; Bichiou et al., 2021). Advances in transcriptome analysis techniques have enabled the identification and characterization of pathways involved in the infection process in both parasites and hosts. These approaches have been lauded due to their advanced effectiveness (Wang et al., 2009; Veras and Bezerra de Menezes, 2016).

In this study, the use of RNA-seq data from infected macrophages with *L. major* and *L amazonensis* allowed the identification of functions related to changes in cell cycle, stress response, DNA repair mechanisms, protein modification processes, and diversification of immunological receptors. These findings could be associated with alterations to macrophage suppression, favoring the parasite’s survival due to the capacity to alter signaling pathways of the macrophage in order to establish the infection (Ghedira et al., 2011; Liu and Uzonna, 2012; Ovalle-Bracho et al., 2015; Fernandes et al., 2016). Furthermore, these alterations in macrophage biological processes could differ according to the infective species (Liu and Uzonna, 2012; Ovalle-Bracho et al., 2015). Similar to Zhang and coworkers, the majority of genes found as DE was identified at initial infection time points (4 and 24 hpi) (Zhang et al., 2010).

Considering the immune response in cutaneous leishmaniasis, the presence of Th17 cells is related to the maintenance of the *in situ* inflammatory process due to the Th1/Th2 responses in skin lesion ulceration (Gonzalez et al., 2020). Our results showed that genes involved in the Th17 pathway are overexpressed on macrophages infected with both *Leishmania* species. Some studies have shown that one of the functions of the Th17 response is to kill pathogens *via* IL-17 production. It has also been reported that this pro-inflammatory cytokine could induce the expression of IL-6, IL-8, GM-CSF, and G-CSF (Katara et al., 2012; Katara et al., 2013; Gonçalves-de-Albuquerque et al., 2017). However, the role of Th17 in leishmaniasis is still unclear because, in CL, it is related to exacerbation of the lesions and disease pathology (Banerjee et al., 2016; Gonzalez et al., 2020).

A strategy to help clarify these questions could be the evaluation of the transcriptional profiles involved in this response. The modulation of the transcriptional profile in macrophages after the invasion by a pathogen is dynamic and changes along the course of the infection. At the initial stages, we observed the expression of genes related to immunological regulation; among these, genes that encode transcription factor STAT2, IL-2, and CXCL12 were identified as overexpressed until 24 hpi. These molecules are important for effective inflammatory response during infection, being responsible for inducing T-cell recruitment, proliferation, regulation, and production of cytokines such as type I interferons (IFN-α and IFN-β) (Steen and Gamero, 2012; Ross and Cantrell, 2018; Pol et al., 2019). Similar to other studies, an increase in the expression of CXCL10 and CCL2 was also observed (Rabhi et al., 2012).

It is known that IFN-α and IFN-β are related to the expression of nitric oxide synthase type 2 (NOS2) and production of NO by macrophages, as other studies have shown the importance of this pathway against intracellular infections (Diefenbach et al., 1998; Xin et al., 2008; Cummings et al., 2010). In this context, we hypothesize that the parasite starts the modulation of these genes until the second time-point (24 hpi), resulting in the reduction at third and fourth time-points in order to create a favorable microenvironment to establish the infection, neutralizing part of the macrophage defense machinery. In murine models, an infection caused by *L. amazonensis* showed that amastigotes induce a reduction of STAT-2 phosphorylation and an increase of degradation through parasite proteases (Xin et al., 2008).

Advances in transcriptome approaches allowed the identification of pathways related to the infective mechanisms in parasites and hosts. On the other hand, many of these approaches focus mainly on single genes, not in a global view of the pathogenesis process (Menezes et al., 2013; Castiglione et al., 2016; Lee et al., 2018; Veras et al., 2018). Different analytical tools are available for studying and analyzing next-generation sequencing (NGS) data; one of them was the GRN approach. The construction of these networks offers a measurement of relationships due to the reconstruction of the pathway (Huang et al., 2009; Kuleshov et al., 2016; Han et al., 2017; Jackson et al., 2020). In the network model, the addition of modifications in the TFs STAT1 and STAT3, CEBPβ (CEBPB-CCAAT/enhancer-binding protein beta), and NF-kB (nuclear factor kappa-light-chain-enhancer of activated B cells) caused differences in the intensity of the signal generated. However, they were not sufficient for a complete blockade of the Th17 response induction in infected macrophages when compared to uninfected macrophages. Approximately 30% of inhibition of the full pathway was achieved when compared to uninfected macrophages. Nonetheless, after the inhibition of selected TFs, we observed that they induced a decrease in the expression of CxCL2 and CCL7 in macrophages infected with *L. major.* For *L. amazonensis*, a decrease in CxCL8 and CXCL9 expression levels was also observed. The addition of inhibitory points in NF-kB also causes a decrease in the potential to induce IL-12 and IL-23 levels. Despite this, it is well known that these ILs were associated with a protective role against leishmaniasis (Dayakar et al., 2019). In the predictive model, this decrease could be related to missing genes in the literature review for the model construction. However, even with the inhibition of TFs, the gene expression related to the IL-17 pathway on infected macrophages did not equalize with that on non-infected macrophages.

## Conclusion

The host–parasite interaction of leishmaniasis has enormous relevance in the disease outcome with many factors involved, as gene expression triggered by this interaction until cell recruitment and activation was due to cytokine and chemokine production and signalization. In this context, GRN is a suitable tool for managing this ocean of factors, specific points for evaluation, and attribute values in a foreseen scenario of dynamic interactions. The GRN computational modeling built in this study was able to predict how the signal (modulation of expression promoted by the alterations) flows through the network. Furthermore, our study was able to follow across determined kinetics the gene expression profile of macrophages infected with two different *Leishmania* spp. Specifically, we were able to predict the following: a) a set of TFs that can cause changes in the gene expression profile; b) a set of genes ranked as crucial for the immune modulation of the pathway (increase or decrease of Th17 final response); and c) the IL-17 pathway model for uninfected and infected macrophages, considering the time course of the infection. These promising results encourage us to keep looking for key factors present in other crucial pathways that we believe are involved in the resistance and susceptibility of macrophage infection by leishmaniasis.

## Data Availability Statement

Publicly available datasets were analyzed in this study. These data can be found here: SRA accession number: SRP062278.

## Author Contributions

LG acquired, analyzed, and interpreted the data and wrote the paper. AP acquired, analyzed, and interpreted the data. FM interpreted the data and wrote the paper. AE interpreted the data. MC interpreted the data, wrote the paper, and revised it critically for intellectual content. DR analyzed and interpreted the data. MP interpreted the data, wrote the paper, and revised critically for intellectual content. JR interpreted the data, wrote the paper, and revised it critically for intellectual content. All authors listed have made a substantial, direct, and intellectual contribution to the work and approved it for publication.

## Funding

JR was funded by CNPq project number 310104/2018- and, INOVA-Fiocruz, Grants VPPIS-001-FIO-18-12 and VPPCB-005-FIO-20-2-42. MC was funded by INOVA-Fiocruz, Grant VPPIS-001-FIO-18-8.

## Conflict of Interest

The authors declare that the research was conducted in the absence of any commercial or financial relationships that could be construed as a potential conflict of interest.

## Publisher’s Note

All claims expressed in this article are solely those of the authors and do not necessarily represent those of their affiliated organizations, or those of the publisher, the editors and the reviewers. Any product that may be evaluated in this article, or claim that may be made by its manufacturer, is not guaranteed or endorsed by the publisher.
