# Supplementary figures and images for: Expression Profile of Genes Related to the Th17 Pathway in Macrophages Infected by Leishmania major and Leishmania amazonensis: The Use of Gene Regulatory Networks in Modeling This Pathway
Source: Front Cell Infect Microbiol. 2022 Jun 14;12:826523. doi: 10.3389/fcimb.2022.826523 (PMC9239034; doi:10.3389/fcimb.2022.826523)

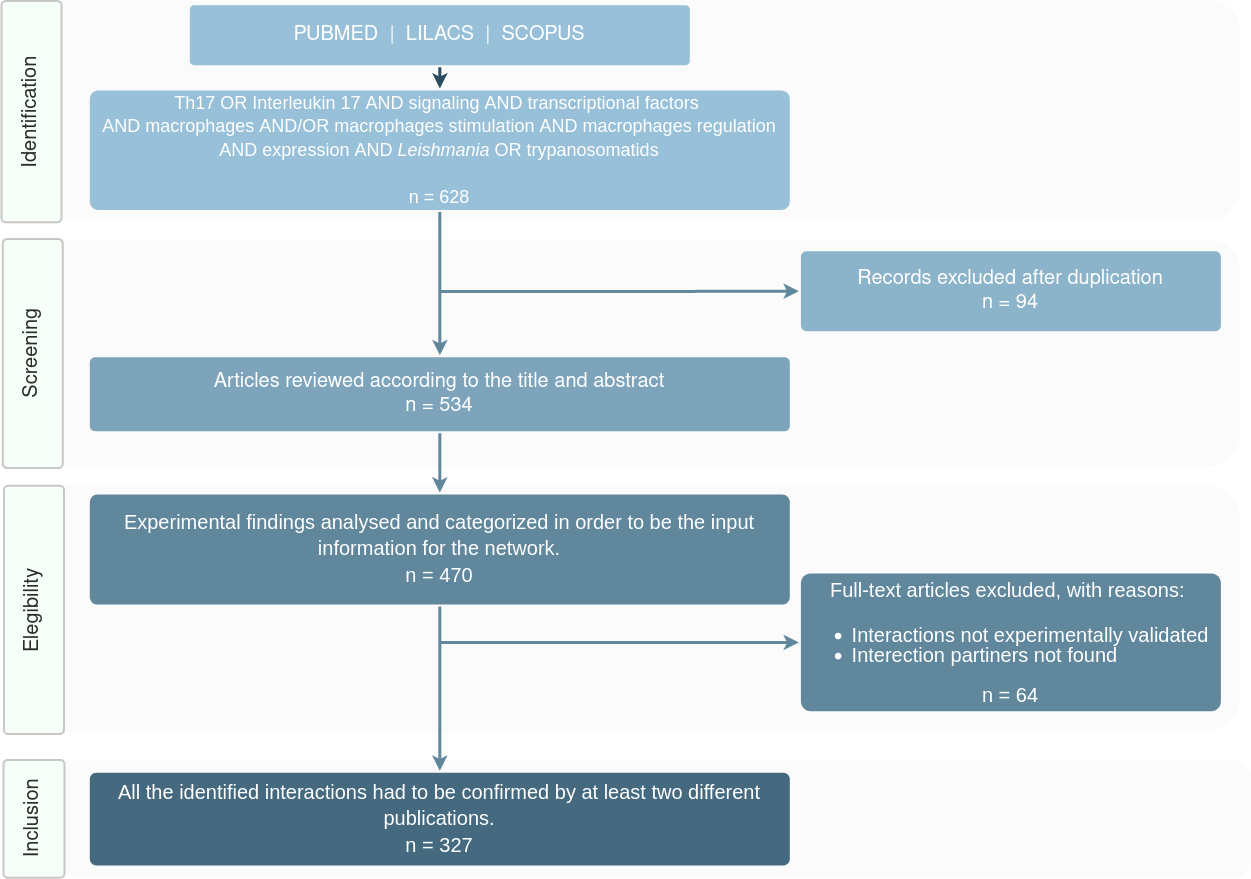

Supplement: Supplementary file 1 [file Image_1.tiff]
